# An improved simple method for the identification of Mycobacteria by MALDI-TOF MS (Matrix-Assisted Laser Desorption- Ionization mass spectrometry)

**DOI:** 10.1038/s41598-019-56604-7

**Published:** 2019-12-27

**Authors:** Adela Alcolea-Medina, M. T. Cabezas Fernandez, N. Montiel, M. P. Luzón García, C. Delamo Sevilla, Nathan North, M. J. Martínez Lirola, Mark Wilks

**Affiliations:** 1 Microbiology, Division of Infection, Barts Health NHS Trust, 80 Newark St, E1 2ES London, UK; 2 C.H.U. Torrecárdenas Almería, Calle Hermandad de Donantes de Sangre, s/n, 04009 Almería, Spain; 3 A.S.H. Costa del Sol, Málaga A-7, Km 187, 29603 Marbella, Málaga Spain; 4 Agencia Publica Sanitaria Poniente, Almeria, Spain; 5 Immunobiology, Blizard Institute, Barts and the London School of Medicine and Dentistry, Queen Mary, University of London, London, UK

**Keywords:** Clinical microbiology, Infectious-disease diagnostics

## Abstract

The aim of this study was to establish a simple method for the rapid identification of Mycobacteria species by MALDI-TOF (Matrix-Assisted Laser Desorption/Ionization-Time of Flight Mass spectrometry) using the Bruker MALDI-TOF Biotyper system (Bruker Daltonik, Bremen, Germany). A multicentre, prospective, and single blind study was performed in three European Hospitals, two Spanish and one UK hospital from May to August 2018. The BD BACTEC MGIT (Becton Dickinson, Berks, UK) liquid culture system was used in all three centres for the growth of Mycobacteria. When signal positive, tubes were removed from the analyser and in addition to standard laboratory procedures were subcultured on blood agar plates for MALDI-TOF analysis. Plates were incubated aerobically for 1 to 7 days at 37 °C and inspected every day. Once any growth was visible, it was transferred to the steel target plate, overlaid with 1 μl of neat formic acid and 1 μl HCCA matrix (alpha hydroxyl 4 cinnamic acid), and analysed in a Bruker Biotyper MALDI-TOF. Results given by MALDI-TOF were compared with the reference methods used for identification in the different centres. At two Spanish hospitals, identification by MALDI-TOF was only attempted on presumptive non-tuberculosis mycobacteria (NTM) and the results were initially compared with the results obtained by a commercial reverse hybridisation assay, GenoType CM/AS (Hain Lifescience, Tübingen, Germany). At the UK Hospital, identification of any presumptive mycobacteria was attempted and compared with the results obtained by whole genome sequencing (WGS). Overall in 142/167 (85%) of cases the identifications obtained were concordant; all *Mycobacterium tuberculosis* (MTB) isolates 43/43 (100%), 57/76 (75%) of the rapid growing nontuberculous mycobacteria (NTM), and 42/48 (85%) slow growing NTM tested were identified correctly. We report a new, easy, cheap and quick method for isolation and identification of *Mycobacterium spp*. without the need for additional steps or equipment and this method is in routine used in all three centres.

## Introduction

Rapid identification of mycobacterial infections and antimicrobial resistance detection remain as one of the biggest challenges for the microbiology lab. The turnaround time for definitive identification, is typically dependent on the services of an external Reference lab, which may lead to long delays. Due to the increasing number of drug resistant MTB and NTM infections, the rapid differentiation of MTB from other Mycobacteria is critical to administer appropriate treatment and establish effective public health interventions^1^.

Nucleic acid amplification assays are excellent tools for direct identification of mycobacteria in clinical specimens^2^. All of them work well for AFB (Acid-Fast Bacillus) positive specimens which are smear positive but are less effective in paucibacillary patients.

In contrast, there are very few commercial assays for direct detection of the diverse range of non NTM species in clinical specimens, therefore, it is necessary to use in-house tests or wait to have the NTM strain in cultivation for definitive identification. It may take 4–6 weeks or longer to identify “slow growing” mycobacteria^2^.

Once cultured, there are several commercial molecular‐based assays for definitive identification of Mycobacteria using a combination of PCR and a method of detecting the PCR product(s) by visualizing the products of hybridization to membrane bound probes for example INNO-LiPA MYCOBACTERIA v2 (LiPA Innogenetics, Ghent, Belgium) and GenoType CM/AS (Hain Lifescience, Tübingen, Germany).

However, these methods are expensive and time consuming and most of them require post‐amplification procedures to distinguish between mycobacterial species. In addition most of these tests are not designed for testing of single isolates, requiring the accumulation of a minimum number of isolates before testing to be cost effective. These factors may contribute to even longer delay in the identification of mycobacteria. Whole genome sequencing has been described as a good alternative for identification and detection of resistance genes directly from samples which may considerably reduce turnaround time but is an expensive technique which requires highly trained staff^3^.

Phenotypic identification of mycobacteria by MALDI-TOF mass spectrometry is better suited to the identification of single or low numbers of isolates. It is far simpler and cheaper than whole genome sequencing (WGS) or any commercial nucleic acid amplification test making it suitable for mycobacteria identification in most routine laboratories which have access to a MALDI-TOF mass spectrometer.

Bruker has developed new software in recent years for *Mycobacterium* identification and validated a specific extraction method for mycobacteria extract method (MycoEX)^4^. The original Bruker’s Mycobacteria extract method was validated for liquid or colonies taken from solid samples, and took around two hours to be performed. Since then it has undergone several modifications, most of them based on cell disruption, adding sonication or bead-beating steps for liberating mycobacterial protein for analysis to improve score values and identification^5^.

We introduce a new and simple technique for Mycobacterium identification with MALDI-TOF, which was evaluated in three hospitals in two different European countries, Hospital Universitario Torrecárdenas (H1-SP), Almeria (Spain), Empresa Pública Hospital de la Costa del Sol, Marbella-Málaga (Spain) (H2-SP) and Barts Health NHS Trust, London (UK) (H3-UK).

## Methods

The BD BACTEC MGIT (Becton Dickinson, Berks, UK) liquid culture system was used in all three centres. Tubes are continuously monitored and oxygen depletion by growing bacteria leads to a change in fluorescence which is signaled automatically. When signal positive, tubes were removed, and examined for the presence of AFB and cord formation. From May to August 2018, a total of 167 signal positive tubes were subcultured on blood agar plate (Oxoid) and incubated under ambient air for 1 to 7 days at 37 °C At centres H1-SP and H2-SP only presumptive non-tuberculosis mycobacteria (NTM) were selected for MALDI-TOF identification (*n* = 99). These did not show cord-formation in an auramine stain and were negative in an immunochromographic test using anti-MPB64 monoclonal antibodies (BD MGIT TBc), a heat killing step was not performed. At H3-UK all the mycobacteria growing on blood agar (*n* = 68) were analysed regardless of their appearance by Ziehl-Neelsen (ZN) staining in the positive BD BACTEC MGIT hence a heat killing step was performed.

In all centres, the blood agar plate’s subcultures were inspected every day in a Class 1 biological safety cabinet, once growth was visible; a disposable plastic loop was used to place it on two wells of the steel target plate. The spotted wells were overlaid with 1 μl of neat formic acid and allowed to dry before adding matrix. 1 μl α-cyano-4-hydroxycinnamic acid (HCCA) matrix dissolved in acetonitrile 50%, water 47.5% and trifluoroacetic acid 2.5% to a concentration of 10 mg/ml was added, allowed to dry and analysed by MALDI-TOF.

The IVD (*in vitro* diagnostic device) certified MALDI Biotyper instrument (Bruker Daltonik, Bremen, Germany) was used for identification in all centres. The results were analysed by two different methods. At H1-SP and H2,-SP the standard Bruker BDAL^™^ library (MBT 6903 MSP Library V6) was used, this library has minimum acceptable identification score value of 1.50. The identification was accepted when a score of 1.50 or higher was obtained and when the identification matched with at least five of the ten top species identifications provided by MALDI-TOF were identical. At H3-UK the specific mycobacterial library (Mycobacteria MBT identification library 4.0) with minimal acceptable score value of 1.60 was used. The MBT Mycobacteria Software Module also uses an adapted data acquisition and analysis methods compared to the standard MALDI Biotyper method.

The results given by MALDI-TOF were compared with the reference methods in routine use for identification at the different centres. At Spanish Centres H1-SP and H2-SP, the reference method was the Hain GenoType CM (Common Mycobacteria) and AS (Additional species) tests (Hain Lifescience, Tübingen, Germany). The CM and AS kits identify mycobacteria using reverse hybridisation of PCR amplicons to membrane‐bound probes covering the species‐specific variable regions of the 23S target gene. When the identification obtained by this test and MALDI-TOF were different, mycobacteria were further identified by 16S rRNA gene sequencing or use of the Hain GenoType NTM-DR kit which is intended to differentiate between three species within the *Mycobacterium avium* complex, namely, *Mycobacterium avium*, *Mycobacterium intracellulare*, and *Mycobacterium chimaera*. At centre H3-UK, the MALDI-TOF identifications were compared retrospectively with the results obtained by whole genome sequencing provided by the National Mycobacterial Reference Laboratory South, Public Health England.

At centre H3-UK, the MALDI-TOF MSP 96 Polished Steel Targets was spotted with the strains for identification and bacteria killed by heating for 30 min at 95 °C on a slide fixing hot plate (RA Lamb Slide Drying Hotplate, ThermoFisher, Basingstoke, UK). Slides were removed, allowed to cool for 1 minute and 1 μl neat formic acid added. This was allowed to dry and the well overlaid with 1 μl of α-cyano-4-hydroxycinnamic acid (HCCA) matrix at a concentration of 10 mg/ml. The efficacy of heat killing was shown by rubbing the surface of the steel plate with a saline moistened swab which was then smeared on a blood agar plate, Lowenstein-Jensen (LJ) slope and finally inoculated into a Mycobacteria Growth Indicator Tube (MIGIT) and incubated in the BD BACTEC MGIT automated system (Becton Dickinson, Wokingham, Berkshire). All cultures were incubated at 37 °C for six weeks. No growth was detected after the incubation period.

### Ethical approval

There was no requirement for ethical approval in this laboratory based study, no patient identifiers were reported in any of the study centres, no results were used in patient management.

### Informed consent

There was no requirement for informed consent in this laboratory based study.

## Results

One hundred and sixty-seven positive specimens were subcultured on blood agar and the results of identification by MALDI-TOF and the reference methods are summarised in Table 1. MALDI-TOF identification resulted in 42/48 (87%) of the NTM rapid growing, 57/76 (75%) of NTM slow growing and 43/43 (100%) for MTB.(See Table 2) Twenty-four specimens were not identified by MALDI-TOF (Table 3). At centres H-SP1 and H-SP2, the score values obtained with the MALDI-TOF were a mean of 1.89, median 1.87 a and a range of 1.5–2.39. At Centre H3-UK the score values with the Mycobacteria library obtained from all identifications were a mean of 1.82, median of 1.8 and a range of 1.61–2.31.

**Table 1:** Results of identifications and reference methods used.

Centre	H1-SP and H2-SP	H3-UK
Primary reference method used	GenoType CM/AS	WGS
Further reference method used if discrepant results were obtained between the primary reference method and the MALDI-TOF identification	16S rRNA gene sequencing/ GenoType NTM ID	N/A
MALDI-TOF library used	Bruker BDAL™ library (MBT 6903 MSP Library V6)	Mycobacteria MBT™ identification library V4.0
Number of samples	99	68
MALDI-TOF ID	76 (77%)	66 (97%)
Discrepant results	5 (5%)	None
No identification obtained with MALDI-TOF	23 (23%)	1 (1.5%)
Identification obtained by reference method	99 (100%)	66 (97%)
No identification obtained by reference method	None	2 (3%)
More detailed information obtained by ref method	None	9 (13%)

**Table 2:** Range of species and number of isolates identified by MALDI-TOF in this study.

Rapid growers	Slow growers
*M. abscessus* (24)	*M. avium* (12)
*M. chelonae* (4)	*M. celatum* (1)
*M. elephantis* (1)	*M. gordonae* (9)
*M. farcinogenes* (2)	*M. chimaera-intracellulare group* (27)
*M. fortuitum* (8)	*M. kansansii* (3)
*M. peregrinum* (1)	*M.lentiflavum* (1)
*M. porcinum* (2)	*M. simae* (3)
*M. abscessus* (24)	*M. tuberculosis complex* (43)
*M. chelonae* (4)

**Table 3:** Mycobacteria not identified by MALDI-TOF.

Identification obtained by reference method (*n*)	Reference method used
*M. gordonae* (4)	Genotype CM-AS
*M. avium* (8)	Genotype CM-AS
*M. lentiflavum*(1)	Genotype CM-AS
*M. chelonae* (1)	Genotype CM-AS
*M. fortuitum* (1)	Genotype CM-AS
*M. abscessus* (2)	Genotype CM-AS
*M. intracellulare* (6)	Genotype CM-AS
*Mycobacterium sp*.	WGS

Results with Genotype CM-AS and MALDI-TOF were discrepant in 5 samples, but were concordant with MALDI-TOF identification when GenoType NTM-DR or 16S sequencing were performed. Whole genome sequencing provided a more precise identification than MALDI-TOF results in 9 cases. Thus isolates identified as *M chimera/intracellulare* group by MALDI-TOF was identified as either *M. chimaera* or *M*.*intracellulare* by WGS. One isolate was identified as *M. porcinum* by MALDI-TOF although identified as the closely related species *M. fortuitum* by WGS (see Table 4).

**Table 4:** Results which were discrepant between those obtained by MALDI-TOF and the reference method.

Identification obtained by reference method (*n*)	Reference method used (Second reference method used if required)	MALDI-TOF Identification
*M fortuitum* (1)	WGS	*M. porcinum*
*M. fortuitum* (1)	WGS	*Tsukamurella paurometabola*
*M. africanum* (1)	WGS	*M. tuberculosis complex*
*M. chimaera* (5)	WGS	*M. chimaera-intracellulare group*
*M. intracellulare* (1)	Genotype CM-AS (Genotype NTM-DR)	*M. chimaera-intracellulare group*
*M. avium/M. chimaera* (1)	Genotype CM-AS (Genotype NTM-DR)	*M. chimaera-intracellulare group*
*M. fortuitum/M. farcinogenes* (2) *Mycobacterium chelone/M.abscessus* (1)	Genotype CM-AS (16S RNA) Genotype CM-AS (Genotype NTM-DR)	*M. farcinogenes M. abscessus*

One of the cultures was contaminated with *Tsukamurella paurometabola* and this was identified by MALDI-TOF; WGS sequencing was performed twice after detection of an unidentified contaminant and at the second attempt *M. fortuitum* was identified.

WGS failed to identify 2 samples despite repeated attempts. In one case this was identified by MALDI-TOF as *M. peregrinum* but in the other case this could not be identified by MALDI-TOF either.

After subculture on blood agar media, the time taken to achieve visible growth was typically 5 days for the slow growing Mycobacteria and for the rapid growing Mycobacteria 2 days.

## Discussion

We describe an easy, cheap and quick method for *Mycobacterium spp*. identification with MALDI-TOF biotyper from positive MGIT cultures positive which have been subcultured on blood agar and inactivated. 97% of the isolates were identified to the species level. Previously, we could identify only 35% of isolates using the Mycobacteria extraction method (MycoEX) proposed by Bruker which includes several steps of centrifugation and vortexing and took around 2 hours to perform for several samples. The MycoEX method has been used for identification of *Mycobacterium spp*. in solid culture instead of liquid culture and higher rates of identification were obtained^6^. However, this could lead to delays in identification for a week and it is more laborious than the method described in our study. Subsequently a modified MycoEX method including sonication and bead-beating was introduced and reached 68.2% successful identification in our hands. Other studies have reported similar percentages of identification with this method^5,7^.

Another method for the identification of *M. tuberculosi*s from MGIT positive bottles using the VITEK MS (bioMérieux, Marcy l’Étoile, France) MALDI-TOF system has been described. The rates of identification are lower than the method describe using the Bruker Mycobacteria library and the method is more laborious involving centrifugation and lysis^8^.

The isolation of organisms of the *M. tuberculosis* complex typically utilises egg-based medium, however isolation of *M. tuberculosis* on blood agar has been reported previously in several studies. The time required to isolate *M. tuberculosis* has been reported to be around one week^9,10^. *M. tuberculosis* growing directly on blood agar plate, subcultured from a positive MGIT culture tube, is reported for the first time to our knowledge. In our study, some *Mycobacterium tuberculosis* isolates were identified after only 24 h of incubation. Laboratories who adopt this method must be aware of the possibility of *M. tuberculosis* complex growing after such a short space of time and plates should not be examined on the open bench^11^.

MALDI-TOF identified bacteria closely related to *Mycobacteria* such as *Tsukamurella sp*, *Nocardia spp*, *Gordonia sp*, *Methylobacterium organophilum* and *Actinomyce*s which may also survive the action of sodium hydroxide used to decontaminate sputum specimens and so will grow and be be recovered on the blood agar plate^12^ . Some cases of misidentification have been reported using the Hain Genotype Molecular Genetic Assay^13^. In this study one of the samples was overgrown by *Tsukamurella* which did not allow the identification of *M. fortuitum* identified by WGS after two attempts.

Generally the reference method provided a more detailed identification such as the differentiation between *M. chimaera* and *intracellulare* and within the MTB complex. In some cases the results provided by MALDI-TOF were more detailed than that provided by the reference method, such as *M. porcinum* which was identified as a *M. fortuitum* with WGS^14^.

We propose a new method for isolation and identification of *Mycobacterium spp*. which it is easy and adds little extra cost. This should prove especially useful for smaller laboratories which do not have ready access to methods for the identification of *Mycobacterium spp*. other than MALDI-TOF and are dependent on sending isolates to external reference labs with the inevitable delay. The new method described allows identification of a much larger number of species at no extra cost compared to more complex expensive methods such as INNO-LiPA^TM^ MYCOBACTERIA which can only identify 16 species of Mycobacterium as even with the standard Bruker MALDI Biotyper library the percentage identification was 77%.

As expected, the higher number of identifications was obtained using the specific Mycobacteria MBT™ identification library 4.0 giving an increase of 20% of identifications compared to the standard Mycobacteria library.

At H3, this Mycobacteria MALDI-TOF identification provided a much quicker result for identification reducing the time around time (TAT) in our laboratory from 17.97 (44–98) to 3.6 (1–7) days. The UK Standards for Microbiology Investigations (UK SMIs) recommends slides prepared from positive mycobacterial cultures are heat fixed by heating to 65–75 °C for 10 minutes before removal from a safety cabinet, the method described here for MALDI-TOF preparation considerably exceeds this and as such this method has no greater risk than staining and reading a ZN smear^15^.

The time taken to perform this technique is around 40 minutes for several samples, after eliminating all centrifugation, vortexing and sonication steps. This method has now been successfully implemented in our laboratories leading to a significant reduction in the time between detection and identification.
